# Patient-reported gastrointestinal symptoms following surgery for gastric cancer and the relative risk factors

**DOI:** 10.3389/fonc.2022.951485

**Published:** 2022-09-14

**Authors:** Rui Xu, Qiong Gu, Shuomeng Xiao, Ping Zhao, Zhi Ding

**Affiliations:** Department of Gastrointestinal Surgery, Sichuan Cancer Hospital and Institute, Sichuan Cancer Center, School of Medicine, University of Electronic Science and Technology of China, Chengdu, China

**Keywords:** surgery, gastrectomy, gastric cancer, symptom, risk factor

## Abstract

**Purpose:**

The study aims to assess the incidence of short-term patient-reported postoperative gastrointestinal symptoms (PGISs) after gastric cancer surgery and explore the relative risk factors for the symptoms.

**Methods:**

Patients with radical gastrectomy were included for this retrospective and observational study. Symptoms extracted from the MD Anderson Symptom Inventory Gastrointestinal Cancer Module (MDASI-GI) were collected in postdischarge week (PDW) 1 and postoperative month (POM) 1. The distributing states of symptoms were analyzed in PDW1 and POM1. Logistic regression models were used to identify risk factors for PGISs.

**Results:**

Among 356 patients with complete interviews, 156 (43.8%) patients reported abdominal distention in PDW1, which was significantly higher than patients in POM1 [103 (28.9%), *p* < 0.001]. Pain (15.2% vs. 9.8%), dysphagia (5.6% vs. 7.0%), diarrhea (3.7% vs. 3.4%), and vomiting (2.5% vs. 2.8%) had no significant differences between PDW1 and POM1. Logistic models found that risk factors for PGISs were total gastrectomy [odds ratio (OR): 1.948; 95% CI: 1.097–3.459; *p* = 0.023] and disturbed sleep (OR: 3.116; 95% CI: 1.831–5.303; *p* < 0.001) in PDW1 and female gender (OR: 1.726; 95% CI: 1.071–2.782; *p* = 0.025), total gastrectomy (OR: 1.729; 95% CI: 1.055–2.834; *p* = 0.030), and disturbed sleep (OR: 3.533; 95% CI: 1.757–7.106; *p* < 0.001) in POM1.

**Conclusions:**

The main symptom after gastric cancer surgery was abdominal distention. The relative risk factors for gastrointestinal symptoms after gastric cancer surgery were total gastrectomy and disturbed sleep. Timely symptom intervention may improve the quality of life of postgastrectomy patients.

## Introduction

Gastric cancer (GC) is the fifth most common type of cancer and the third leading cause of cancer deaths worldwide (1). In China, GC is still an upper gastrointestinal malignancy with high morbidity and mortality (2). At present, radical gastrectomy is the first-line treatment for GC that can prolong patients’ survival time (3, 4). As surgery for GC gradually shifts toward minimally invasive approaches that can minimize trauma and postoperative complications and accelerate recovery (5, 6), postoperative gastrointestinal symptoms (PGISs) such as abdominal distention, pain, and vomiting continue to exist that affect quality of life (7).

In recent years, more attention is being given to the patient’s subjective feelings as part of the recovery process (8). However, postoperative symptoms are not directly observable unless the patient reports them to the clinician (9). Patient-reported outcome (PRO) report is an important tool to assess the patient’s physical health, functional status, and posttreatment feeling without interpretation bias from the medical personnel (10, 11). Many studies assumed that PROs reflect more accurately on the patient’s perspective and assist clinicians to improve the quality of life of patients (12, 13).

PGIS is one of the common postoperative symptoms of GC. Gastrointestinal symptoms could increase patient distress and contribute to changes in functional status, treatment failure, depression, anxiety, and poor quality of life (14). In order to promote postoperative recovery and improve patients’ quality of life, this study aims to understand short-term patient-reported PGISs and explore relative risk factors of PGISs.

## Methods

### Participants

This study included patients with GC who underwent radical gastrectomy with D2 lymphadenectomy in the Department of Gastrointestinal Surgery of Sichuan Cancer Hospital in China between January 2020 and December 2021. Informed consent was taken from all of the participants in this study. The inclusion criteria were as follows: 1) age 18 year and above; 2) GC tumor–node–metastasis (TNM) stage c/pT1-4NxM0; 3) under standard radical gastrectomy (total, proximal, and distal gastrectomy). Exclusion criteria included: 1) previous abdominal radiotherapy; 2) preoperative chemotherapy; 3) pulmonary, cardiovascular, and renal disease; 4) mental illness; 5) diabetes.

### Data collection

The electronic medical record system provided age, gender, surgical data, and TNM stage according to the eighth edition of “TNM Classification of Malignant Tumors” by the American Joint Committee on Cancer/Union for International Cancer Control (15). The double-tract reconstruction was used in proximal gastrectomy. Roux-en-Y anastomosis of the esophagus and jejunum was performed in total gastrectomy. Multiple anastomotic methods were applied to distal gastrectomy, such as Billroth I, Billroth II, and Billroth II plus Braun anastomosis and Roux-en-Y anastomosis of the remnant stomach and jejunum. PGISs and sleep status were reported by patients, and each patient was interviewed by a healthcare provider who was trained in qualitative interviewing. The interviews were qualitative one-to-one interviews, which were conducted postdischarge week (PDW) 1 and postoperative month (POM) 1. Each interview lasted for 20 min per patient. PGISs (pain, abdominal distention, diarrhea, vomiting, dysphagia) and disturbed sleep were asked during the interview. All of the symptoms were extracted from the MD Anderson Symptom Inventory Gastrointestinal Cancer Module (MDASI-GI), a valid reliable questionnaire for assessing symptom severity and interference with function in gastrointestinal cancer (16, 17).

### Data analysis

Demographic data, clinical data, and symptoms were presented as numbers and percentages. The t test and chi-square test were used to examine the quantitative and categorical data, respectively.

We performed univariate analyses using gender (women vs. men), approach of operations (open surgery vs. laparoscopic surgery), type of gastrectomy (total vs. proximal vs. distal), TNM stage (stages I–II vs. stage III), and sleep status (disturbed vs. non-disturbed), and PGISs were taken as the dependent variable. Those variables with *p*-values ≤0.05 were entered into the multivariate logistic analyses to determine the risk factors for PGISs.

Statistical analyses were performed using SPSS 20.0 software (IBM Corp., Armonk, NY, USA). A two-sided *p*-value <0.05 was considered significant.

## Results

### Patient characteristics

A total of 356 cases of postoperative patients with GC were involved in the study. There were 249 (69.9%) men and 107 (30.1%) women, and over 50% of the patients were aged ≤60 years (50.3%). Fifty (14%) patients underwent open surgery, and 306 (86%) patients underwent laparoscopic surgery. Of patients included in the study, 215 (60.4%) patients were staged with TNM I–II, and 141 (39.6%) patients were staged with TNM III. Baseline demographic and clinical features are summarized in **Table 1**.

**Table 1:** Demographics and clinical characteristics.

Variables	Number (%)
Age (years)	
≤60	179 (50.3)
>60	177 (49.7)
Gender	
Men	249 (69.9)
Women	107 (30.1)
Surgical approach	
Open surgery	50 (14.0)
Laparoscopic surgery	306 (86.0)
Type of gastrectomy	
Total	100 (28.1)
Proximal	30 (8.4)
Distal	226(63.5)
T stage	
T1	90 (25.3)
T2	59 (16.6)
T3	108 (30.3)
T4	99 (27.8)
N stage	
N0	155 (43.5)
N1	64 (18.0)
N2	69 (19.4)
N3	68 (19.1)
TNM stage	
I–II	215 (60.4)
III	141 (39.6)

### Postoperative gastrointestinal symptoms

**Figure 1:**
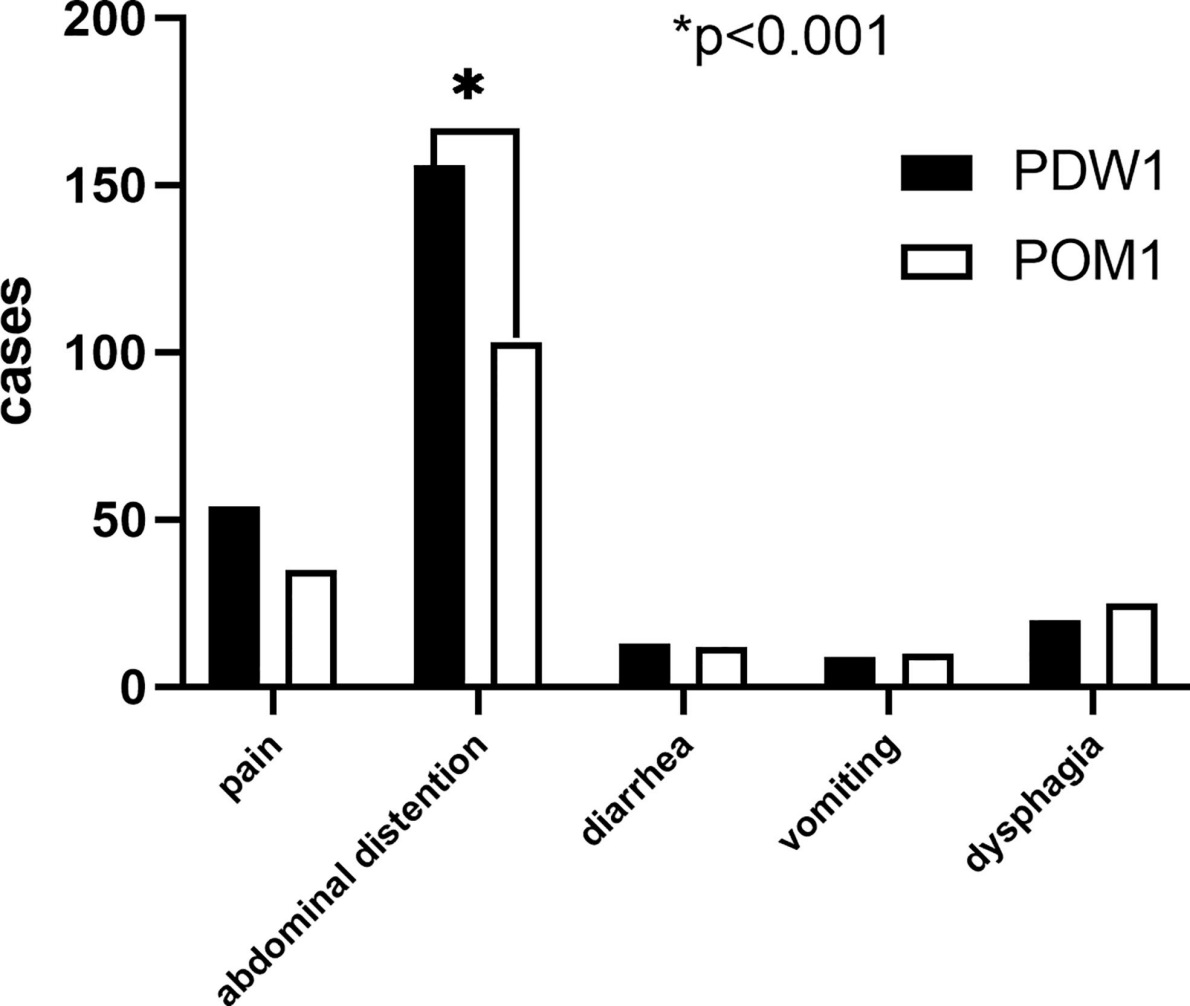
Frequency of postoperative gastrointestinal symptoms in the 356 patients. * *p* < 0.001.

A total of 252 (70.8%) patients reported PGISs in PDW1, comprising abdominal distention (156, 43.8%), pain (54, 15.2%), dysphagia (20, 5.6%), diarrhea (13, 3.7%), and vomiting (9, 2.5%). Of the 356 patients, 185 (52.0%) patients reported PGISs about abdominal distention (103, 28.9%), pain (35, 9.8%), dysphagia (25, 7.0%), diarrhea (12, 3.4%), and vomiting (10, 2.8%) in POM1. The ratio of abdominal distention symptom reported in POM1 was lower than that in PDW1 (28.9% vs. 43.8%, *p* < 0.001). However, similar numbers of other PGISs were reported at two points in time (**Figure 1**).

### The relations between postoperative gastrointestinal symtoms and clinical features

In POM1, the ratio of PGISs in men was less than that in women (48.2% vs. 60.7%, *p* < 0.001). In **Table 2**, compared with patients who underwent distal gastrectomy, the ratio of dysphagia was significantly higher in patients with total gastrectomy in PDW1 and POM1 (all *p* < 0.001) and the ratio of dysphagia was also significantly higher in patients with proximal gastrectomy in PDW1 (*p* = 0.002) and POM1 (*p* = 0.003).

**Table 2:** The relations between PGISs and clinical characteristics.

Variables	PDW1						POM1					
	Non-PGISs	Abdominal distention	Pain	Diarrhea	Vomiting	Dysphagia	Non-PGISs	Abdominal distention	Pain	Diarrhea	Vomiting	Dysphagia
Age (years)												
≤60	47 (26.3)	80 (44.7)	28 (15.6)	8 (4.5)	7 (3.9)	9 (5.0)	77 (43.0)	57 (31.8)	20 (11.2)	6 (3.4)	8 (4.5)	11 (6.1)
>60	57 (32.2)	76 (42.9)	26 (14.7)	5 (2.8)	2 (1.1)	11 (6.2)	94 (53.1)	46 (26.0)	15 (8.5)	6 (3.4)	2 (1.1)	14 (7.9)
Gender												
Men	78 (31.3)	108 (43.4)	37 (14.9)	9 (3.6)	5 (2.0)	12 (4.8)	129 (51.8)**^#^**	71 (28.5)	19 (7.6)	9 (3.6)	5 (2.0)	16 (6.4)
Women	26 (24.3)	48 (44.9)	17 (15.9)	4 (3.7)	4 (3.7)	8 (7.5)	42 (39.3)	32 (29.9)	16 (15.0)	3 (2.8)	5 (4.7)	9 (8.4)
Surgical approach												
Open surgery	16 (32.0)	19 (38.0)	8 (16.0)	2 (4.0)	2 (4.0)	3 (6.0)	24 (48.0)	14 (28.0)	6 (12.0)	2 (4.0)	1 (2.0)	3 (6.0)
Laparoscopic surgery	88 (28.8)	137 (44.8)	46 (15.0)	11 (3.6)	7 (2.3)	17 (5.6)	147 (48.0)	89 (29.1)	29 (9.5)	10 (3.3)	9 (2.9)	22 (7.2)
Type of gastrectomy												
Total	21 (21.0)	45 (45.0)	14 (14.0)	5 (5.0)	1 (1.0)	14 (14.0)*	39 (39.0)	29 (29.0)	8 (8.0)	7 (7.0)	2 (2.0)	15 (15.0)*
Proximal	8 (26.7)	11 (36.7)	5 (16.7)	1 (3.3)	1 (3.3)	4 (13.3)^†^	14 (46.7)	8 (26.7)	2 (6.7)	0 (0.0)	1 (3.3)	5 (16.7)^‡^
Distal	75 (33.2)	100 (44.2)	35 (15.5)	7 (3.1)	7 (3.1)	2 (0.9)	118 (52.5)	66 (29.2)	25 (11.1)	5 (2.2)	7 (3.1)	5 (2.2)
TNM stage												
l–ll	61 (28.4)	97 (45.1)	35 (16.3)	8 (3.7)	7 (3.3)	7 (3.3)	111 (51.6)	61 (28.4)	22 (10.2)	6 (2.8)	5 (2.3)	10 (4.7)
III	43 (30.5)	59 (41.8)	19 (13.5)	5 (3.5)	2 (1.4)	13 (9.2)	60 (42.6)	42 (29.8)	13 (9.2)	6 (4.3)	5 (3.5)	15 (10.6)

PDW, postdischarge week; POM, postoperative month; PGISs, postoperative gastrointestinal symptoms.

**^#^** p < 0.001 compared with women; *p < 0.001 compared with distal gastrectomy; **^†^** p = 0.002 compared with distal gastrectomy; **^‡^** p = 0.003 compared with distal gastrectomy.

### The relations between postoperative gastrointestinal symtoms and sleep status

As **Table 3** shows, patients with disturbed sleep reported PGISs more than patients with non-disturbed sleep in PDW1 and POM1 (all *p* < 0.001).

**Table 3:** The relations between PGISs and sleep status.

	PDW1		*p*-value	POM1		*p*-value
	Non-PGISs	PGISs		Non-PGISs	PGISs	
Sleep status			**<0.001**			**<0.001**
Disturbed	23 (16.1)	120 (83.9)		12 (24.0)	38 (76.0)	
Non-disturbed	81 (38.0)	132 (62.0)		159 (52.0)	147 (48.0)	

PDW, postdischarge week; POM, postoperative month; PGISs, postoperative gastrointestinal symptoms. The bold values mean that there are statistically significant.

### Risk factors for postoperative gastrointestinal symtoms

The multivariate logistic regression model identified that variables significantly associated with PGISs in PDW1 included total gastrectomy [odds ratio (OR): 1.948; 95% CI: 1.097–3.459; *p* = 0.023], disturbed sleep (OR: 3.116; 95% CI: 1.831–5.303; *p* < 0.001), and female gender (OR: 1.726; 95% CI: 1.071–2.782; *p* = 0.025) and total gastrectomy (OR: 1.729; 95% CI: 1.055–2.834; *p* = 0.030) and disturbed sleep (OR: 3.533; 95% CI: 1.757–7.106; *p* < 0.001) in POM1 (**Table 4**).

**Table 4:** Multivariate logistic regression analysis of risk factors for PGISs.

Relative factors	PDW1	Multivariate analysis			POM1	Multivariate analysis		
	N	OR	95%CI	*P*- value	n	OR	95%CI	*P*- value
Gender								
Women	81	1.299	0.759-2.224	0.340	65	1.726	1.071-2.782	**0.025**
Men	171	Ref.			120	Ref.		
Surgical approach								
Open surgery	34	0.868	0.445-1.695	0.679	26	0.884	0.475-1.647	0.698
Laparoscopic surgery	218	Ref.			159	Ref.		
Type of gastrectomy								
Total	79	1.948	1.097-3.459	**0.023**	61	1.729	1.055-2.834	**0.030**
Partial	173	Ref.			124	Ref.		
TNM stage								
I–II	154	1.182	0.722-1.937	0.506	104	0.727	0.463-1.140	0.165
III	98	Ref.			81	Ref.		
Sleep status								
Disturbed	120	3.116	1.831-5.303	**<0.001**	38	3.533	1.757-7.106	**<0.001**
Non-disturbed	132	Ref.			147	Ref.		

PDW, postdischarge week; POM, postoperative month; PGISs, postoperative gastrointestinal symptoms. The bold values mean that there are statistically significant.

## Discussion

Gastrointestinal symptoms are particularly common after abdominal surgery (18, 19). To our knowledge, this is the first study to alone observe short-term PGISs in GC. Five symptoms extracted from MDASI-GI were reported by patients through interviews. In this study, we found that abdominal distension is the main PGIS (43.8% vs. 28.9%), followed by pain (15.2% vs. 9.8%), dysphagia (5.6% vs. 3.4%), diarrhea (3.7% vs. 3.4%), and vomiting (2.5% vs. 2.8%) in PDW1 and POM1. Furthermore, with the recovery process, abdominal distension symptom was significantly relieved within 1 month after surgery, and other symptoms were also relieved but without statistical differences. Abdominal distension is the most common symptom after GC surgery and often associated with gastrointestinal dysfunction; studies found (18) that operation, analgesia, and inflammatory reaction could lead to the occurrence and development of gastrointestinal dysfunction. Therefore, the proportion of abdominal distension in early postoperative period was high, but with the recovery of gastrointestinal function, its ratio will decrease significantly. Previous studies showed that PGISs could increase the risk of postoperative complications, prolong the time of hospitalization, delay the follow-up adjuvant treatment, and reduce the quality of life (20, 21). PRO can provide the real feeling of patients after surgery, so that clinicians can carry out the necessary intervention to accelerate postoperative rehabilitation and improve the quality of life.

In this study, we found that the incidence of dysphagia symptom was higher in patients with total and proximal gastrectomy. This is consistent with studies from Lee et al. (22) and Choi et al. (23) suggesting that the dysphagia symptom was worse in the total gastrectomy group. Because of the disharmony and inconsistency of contraction and peristalsis between the esophagus and jejunum, patients with total or proximal gastrectomy sometimes suffer from dysphagia when eating. In addition, patients who underwent distal gastrectomy have a normal cardiac structure, so less dysphagia symptom is reported. According to multivariate models, total gastrectomy was considered as a risk factor for PGISs (*p* < 0.05). To reduce the burden of PGISs, surgeons should make the decision of total gastrectomy with caution and give professional dietary guidance after surgery.

The occurrence and development of PGISs are often related to many factors. Ana et al. (24) considered that gender is the influencing factor of gastrointestinal symptoms in patients with advanced cancer. Nolte et al. (25) found that the quality of life also varies by gender. In this study, we found that the ratio of women with PGISs was more than that of men in POM1 (60.7% vs. 48.2%, *p* < 0.001); this result clearly showed that the recovery of the gastrointestinal tract in women was slow after surgery. In fact, women are not only more emotionally sensitive than men but also more likely to think negatively (26, 27). In addition, Chinese male patients may show higher tolerance to their own symptoms due to the influence of traditional culture. Thus, our clinicians should keep in mind that gender will affect PROs and help them adjust their moods.

Moreover, we found that sleep status affected the occurrence of PGISs, and patients with disturbed sleep suffered from PGISs more than those without sleep disturbance (*p* < 0.001). Evidence has indicated that sleep disturbance occurred more frequently among cancer survivors (28), especially in GC (29), which reduces the quality of life and increases the incidence of complications (30). Patients who underwent gastrectomy always have psychosocial distresses such as fear of recurrence, difficulty in resuming social life, and concerns about family and finances (31), which can affect sleep status and then aggravate PGISs. In this study, we found that the risk of increased ratios of PGISs with disturbed sleep was 3.116 times that of patients with non-disturbed sleep in PDW1 (OR: 3.116; 95% CI: 1.831–5.303; *p* < 0.001) and 3.533 times in POM1 (OR: 3.533; 95% CI: 1.757–7.106; *p* < 0.001). Clinically, the sleep status and psychological status of patients are often ignored by surgeons. Therefore, clinicians need to pay more attention to sleep conditioning and psychological comfort.

In order to improve patients’ symptoms and enhance the quality of life of patients, timely intervention is very important. Comprehensive health education was proven to be an effective method, which could markedly improve patients’ quality of life through disease awareness-raising activities, guidance on behavior and lifestyle, rehabilitation management, and psychological counseling (32). Drug therapy is also an option. Previous studies have found that auricular-plaster therapy of traditional Chinese medicine can improve postoperative gastrointestinal dysfunction and the quality of life (33).

This study has several limitations. Firstly, patients and healthcare providers were from the same department; patients’ reported symptoms would be biased. Secondly, few patients underwent open surgery, which may lead us to underestimate the incidence of PGISs. However, minimally invasive surgical approaches to the treatment of GC have become increasingly more common (34). Finally, we only observed the main symptoms; other symptoms reported by patients were not included, which may lead to selection bias. Further research needs to be continued.

## Conclusions

The main symptom after GC surgery was abdominal distention. The relative risk factors for gastrointestinal symptoms after GC surgery were total gastrectomy and disturbed sleep. Timely symptom intervention may improve the quality of life of postgastrectomy patients.

## Data availability statement

The original contributions presented in the study are included in the article/supplementary material. Further inquiries can be directed to the corresponding author.

## Ethics statement

Ethical review and approval was not required for the study on human participants in accordance with the local legislation and institutional requirements. Written informed consent for participation was not required for this study in accordance with the national legislation and the institutional requirements.

## Author contributions

RX and ZD designed the study; QG and SX collected the data; RX drafted the manuscript; PZ, ZD, and SX undertook the statistical analysis. All authors read and approved the final manuscript.

## Funding

This work was supported by the Sichuan Provincial Science and Technology Project (No. 2020YFS0430; No. 2018SZ0263).

## Conflict of interest

The authors declare that the research was conducted in the absence of any commercial or financial relationships that could be construed as a potential conflict of interest.

## Publisher’s note

All claims expressed in this article are solely those of the authors and do not necessarily represent those of their affiliated organizations, or those of the publisher, the editors and the reviewers. Any product that may be evaluated in this article, or claim that may be made by its manufacturer, is not guaranteed or endorsed by the publisher.

## Abbreviations

PGISs, postoperative gastrointestinal symptoms; MDASI-GI, MD Anderson Symptom Inventory Gastrointestinal Cancer Module; PDW, postdischarge week; POM, postoperative month; GC, gastric cancer; PRO, patient-reported outcome; TNM, tumor–node–metastasis.
